# Relationship between emotional coping and depressive symptomatology

**DOI:** 10.1192/j.eurpsy.2021.2008

**Published:** 2021-08-13

**Authors:** I. Delhom, J.C. Melendez, E. Satorres

**Affiliations:** 1 Psychology, Valencian International University, Valencia, Spain; 2 Development Psychology, University of Valencia, Valencia, Spain; 3 Developmental Psychology, University of Valencia, Valencia, Spain

**Keywords:** depressive symptomatology, adaptation, coping, Coping Strategies

## Abstract

**Introduction:**

From the life cycle perspective, the aging is described as the strengthening of adaptive resources and the capacity for recovery or compensation for losses. These skills are grounded in the coping strategies that individuals apply in order to effectively adapt to diverse situations. Emotion-focused, passive coping strategies are considered to be maladaptive in the long term. These strategies are associated with affective disorders, being these phenomena of great impact in older adults.

**Objectives:**

Verify if there is a relationship between emotion-focused coping strategies and depressive symptoms

**Methods:**

The sample was composed of 418 healthy older adults, aged between 60 and 89 years with an average age of 69.67 years and SD = 7.24, 63.6% of the participants are women and the remaining 36.4% are men. The Coping Stress Questionnaire was used to evaluate strategies focused on emotion (Sandín & Chorot, 2003). The Center for Epidemiological Studies-Depression Scale (Radloff & Teri, 1986) was used to evaluate depressive symptoms.

**Results:**

Depressive symptomatology showed significant associations with all emotion-focused strategies: negative self-focus (.339), open emotional expression (.279), avoidance (.202) and religion (113) with a significance level of 0.05.

**Conclusions:**

Emotion-focused coping strategies are associated with depressive symptomatology. Thus, it is considered that the use of these types of strategies in times of change or challenge will not benefit adaptation in the older adult. It is necessary to develop more active coping strategies for prevention in mental health during aging.

**Disclosure:**

No significant relationships.

